# The Magnitude of Neonatal Mortality and Its Predictors in Ethiopia: A Systematic Review and Meta-Analysis

**DOI:** 10.1155/2021/7478108

**Published:** 2021-02-17

**Authors:** Yared Asmare Aynalem, Wondimeneh Shibabaw Shiferaw, Tadesse Yirga Akalu, Abate Dargie, Hilina Ketema Assefa, Tesfa Dejenie Habtewold

**Affiliations:** ^1^College of Health Science, Debre Berhan University, Debre Berhan, Ethiopia; ^2^College of Health Science, Debre Markos University, Debre Markos, Ethiopia; ^3^Department of Epidemiology, University Medical Centre Groningen, University of Groningen, Groningen, Netherlands

## Abstract

**Background:**

Although neonatal death is a global burden, it is the highest in sub-Saharan African countries such as Ethiopia. Moreover, there is disparity in the prevalence and associated factors of studies. Therefore, this study was aimed at providing pooled national prevalence and predictors of neonatal mortality in Ethiopia.

**Methods:**

The following databases were systematically explored to search for articles: Boolean operator, Cochrane Library, PubMed, EMBASE, Hinari, and Google Scholar. Selection, screening, reviewing, and data extraction were done by two reviewers independently using Microsoft Excel spreadsheet. The modified Newcastle-Ottawa Scale (NOS) and the Joanna Briggs Institute Prevalence Critical Appraisal tools were used to assess the quality of evidence. All studies conducted in Ethiopia and reporting the prevalence and predictors of neonatal mortality were included. Data were extracted using Microsoft Excel spreadsheet software and imported into Stata version 14s for further analysis. Publication bias was checked using funnel plots and Egger's and Begg's tests. Heterogeneity was also checked by Higgins's method. A random effects meta-analysis model with 95% confidence interval was computed to estimate the pooled effect size (i.e., prevalence and odds ratio). Moreover, subgroup analysis based on region, sample size, and study design was done.

**Results:**

After reviewing 88 studies, 12 studies fulfilled the inclusion criteria and were included in the meta-analysis. Pooled national prevalence of neonatal mortality in Ethiopia was 16.3% (95% CI: 12.1, 20.6, *I*^2^ = 98.8%). The subgroup analysis indicated that the highest prevalence was observed in the Amhara region, 20.3% (95% CI: 9.6, 31.1), followed by Oromia, 18.8% (95% CI: 11.9, 49.4). Gestational age [AOR: 1.32 (95% CI: 1.07, 1.58)], neonatal sepsis [AOR: 1.23 (95% CI: 1.05, 1.4)], respiratory distress syndromes (RDS) [AOR: 1.18 (95% CI: 0.87, 1.49)], and place of residency [AOR: 1.93 (95% CI: 1.13, 2.73)] were the most important predictors.

**Conclusions:**

Neonatal mortality in Ethiopia was significantly decreased. There was evidence that neonatal sepsis, gestational age, and place of residency were the significant predictors. RDS were also a main predictor of mortality even if not statistically significant. We strongly recommended that health care workers should give a priority for preterm neonates with diagnosis with sepsis and RDS.

## 1. Background

The neonatal period is the first four weeks of a child's life in which changes are very rapid, and many critical events can occur in this period. The death of newborn within the first 28 days of life describes neonatal mortality [1]. Survival of newborn babies had improved significantly through enhanced and specialized care. But still, it is the main reason of under-five death and risk of lifelong risk [2–4]. Globally, the neonatal mortality rate (NMR) declined from 49% to 19%, but slower than the under-five mortality rate (dropped by 60%). Of all deaths of under-five, 40% were also attributed with newborn death [5] in which close to 1 and 2 million deaths occur at the day of birth and in the first week of life, respectively [6]. A review of 20 studies also indicated that the total NMR greatly varied between developed (4 to 46%) and developing (0.2 to 64.4%) countries [7]. Despite this, neonatal mortality shared the highest proportion of under-five deaths worldwide [8–14]. Globally, there are different policies, strategies, and programs which work for the prevention of neonatal morbidity and mortalities [15, 16]. However, neonatal death is still the biggest cause of under-five death worldwide [11, 17–20].

According to the report of the mini-Ethiopian Demographic and Health Survey (EDHS), the neonatal mortality rate accounts for 30% per 1000 live births in Ethiopia [4]. In Ethiopia, different studies have been also conducted to assess the prevalence of neonatal mortality and associated factors [21–25]. The findings of these uneven studies documented that there was a great variability in the prevalence of neonatal mortality across the regions of the country with the lowest prevalence (3.2%) reported by Debelew et al. [26] and the highest prevalence (34.5%) reported by Wesenu et al. [27]. Several factors contribute to the growing burden of neonatal mortality. Some main risk factors include maternal residency [28, 29], history of antenatal care, gestational age, neonatal sepsis, RDS, and asphyxia [24, 30]. With this variation, as far as we know, there has been no systematic review and meta-analysis of studies reporting on the neonatal mortality and its predictors. As such, we believed that synthesizing these studies may fill the gap in the literature. Hence, this study was aimed at synthesizing nationally available evidence on the prevalence of neonatal mortality and the association with different variables in Ethiopia. The findings of this study will be used as an input to policy makers and program planners working in the area of neonatal health.

## 2. Methods

### 2.1. Reporting

The Preferred Reporting Items for Systematic Reviews and Meta-Analyses (PRISMA) guideline [31] was used to report this meta-analysis (Additional file 1 research checklist).

### 2.2. Searching Strategies

The reviewer follows the PRISMA systematic review protocol as a reporting guideline (for the PRISMA checklist, eligible studies for the study were selected in terms of titles alone, abstracts, and then full-text articles, based on inclusion criteria). The Cochrane Library, PubMed, EMBASE, Hinari, African Journals Online, and Google Scholar were systematically searched for articles. These included all fields within records and Medical Subject Headings (MeSH terms). The studies were accessed using the following search terms: “neonatal mortality,” predictors,” “neonatal death,” “newborn,” “prevalence,” “neonatal mortality,” and “Ethiopia.” The search terms were used individually and in combination using “AND” and “OR” Boolean operators. In addition, after identification of included studies, cross-references were searched to identify more eligible studies. The search was guided by PECO: population-neonates (age less than 28 days), occurrence of death within 28 days after delivery.

### 2.3. Inclusion and Exclusion Criteria

Studies with the following major criteria were considered for inclusion. Observational (i.e., cross-sectional, case control and cohort) studies in Ethiopia, which report the prevalence and predictors of neonatal mortality, were included. Articles published in English language were considered as further inclusion criteria. On the other hand, studies which did not report the outcome and articles without full text were excluded. Corresponding authors were approached by email at least twice to access the full text.

### 2.4. Outcome of Interest

PECO:The populations of the study was neonates (age less than 28 days) and the occurrence of death within 28 days after delivery. The main outcome of interest was the prevalence of neonatal mortality in Ethiopia, reported in the reviewed article both as percentage and as a frequency. It was calculated by dividing the number of individuals who have died within the first 28 days of neonatal life to the total number of live birth (sample size) multiplied by 100.

### 2.5. Screening and Data Extraction

Two reviewers (YA and WS) screened titles and abstracts against the inclusion criteria. Then, the full text of the articles was accessed, and independent assessment was carried out by two reviewers based on the predetermined inclusion and exclusion criteria. Discrepancies between the reviewers were resolved through discussion and common consensus of all investigators. Data were extracted from the included papers by three authors(AD, TDH, and TYA) independently from a random sample of 20% of the papers to check consistency; consequently, there was no difference.

### 2.6. Assessment of Study Quality

A structured data abstraction form was constructed in Microsoft Excel. In each abstract and full text of the article, which was considered to be appropriate, a special emphasis was given for clearness of objective, data about the study area, study design, year of publication, study population, sample, size respondent rate, prevalence/incidence of neonatal death, and other useful variables which were recorded (Table 1). The Joanna Briggs Institute Prevalence Critical Appraisal Tool for use in systematic review for prevalence study was used for critical appraisal of studies [32]. Moreover, methodological and other qualities of each article were assessed based on a modified version of the Newcastle-Ottawa Scale for cross-sectional study adapted from Modesti et al. [33].

### 2.7. Data Synthesis and Statistical Analysis

Data were extracted using Microsoft Excel spreadsheet software and imported into Stata version 14 software for further analysis. The pooled effect size with 95% confidence interval of national neonatal mortality rate was determined using a weighted inverse variance random effects model [34]. Heterogeneity across the studies was assessed using the *I*^2^ statistic where 25, 50, and 75% represent low, moderate, and high heterogeneities, respectively [35]. A Funnel plot and Begg's and Egger's tests were used to check publication bias [36]. Moreover, subgroup analyses based on study area (region), study design, and sample sizes were done. Log odds ratios were used to examine the association between mortality and its major predictors.

## 3. Results

### 3.1. Selection of the Studies.

**Figure 1:**
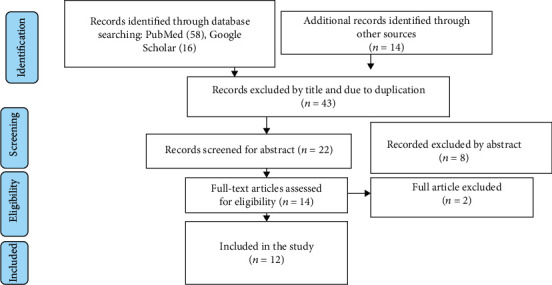
PRISMA flow diagram for showing screening and selection process of duties.

A comprehensive literature search of the database yielded a total of 88 published articles. Of these, 58 articles were retrieved from PubMed, 16 from Google Scholar, and 14 from other sources (EMBASE, Hinari, African Journals Online, and Addis Ababa University digital library). Forty-three articles were excluded after assessing the title and the presence of duplicated publications. About 22 articles were also screened by abstracts, and from these, 8 of the assessed articles were excluded based on the predefined eligibility criteria. Out of them, 14 articles were included and screened for further assessment, of which 12 full-text articles that fulfill the eligible criteria with a total sample size of 12397 neonates were included in the final analysis for the current systematic review and meta-analysis (Figure 1).

### 3.2. Characteristics of the Included Studies

Information about authors, publication year, population, study area, region, study design, outcome, and main results from the selected articles was extracted and is summarized in Table 1. The overall respondent rate was between eighty-three and hundred percent. All studies were done in Ethiopia and published in an indexed journal. Nine of them were cohort studies (both retrospective and prospective), and the remaining three were cross-sectional studies. The studies were conducted in Amhara [21–25], Tigray [37, 38], Southern Nations, Nationalities, and Peoples (SNNP) [28], Oromia [26, 27], and Somali [39] regions. Nine were cohort and three were cross-sectional studies. The sample size of the studies ranged from 485 to 3604 (Table 1).

### 3.3. Prevalence of Neonatal Mortality

**Figure 2:**
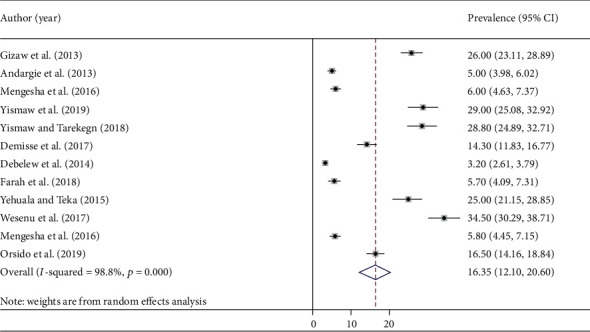
Forest plot of the pooled prevalence of neonatal mortality.

In the current systematic review and meta-analysis, the pooled prevalence estimates of neonatal mortality were described by forest plot. The pooled prevalence of neonatal mortality from the random effects method was found to be 16.3% (95% CI: 12.1, 20.6) (Figure 2).

### 3.4. Subgroup Analysis

Based on the subgroup-analysis result, the highest prevalence (20.3%; 95% CI: 9.6-31.1) was reported in the Amhara region with regard to sample size; the prevalence of neonatal mortality was higher in studies having a sample size < 800 [22.7% (95% CI: 12.8, 32.7)] compared to those having a sample size ≥ 800 [8.9 (95% CI: 4.9, 12.9)] (Table 2).

### 3.5. Assessment of Publication Bias

**Figure 3:**
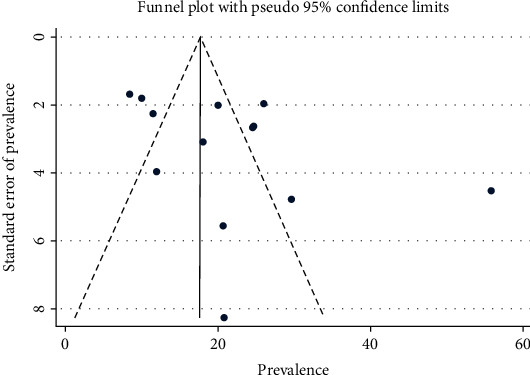
Funnel plot to show the distribution of 12 studies.

The funnel plot was found to be asymmetric (Figure 3), and Egger's and Begg's tests show a significant publication bias at *p* value of ≤0.001.

### 3.6. Investigation of Heterogeneity

To observe the possible causes of difference across studies, we conducted a metaregression analysis using publication year and sample size. But the result showed that there is no significant heterogeneity (Table 3).

### 3.7. Predictors of Neonatal Mortality

#### 3.7.1. Gestational Age

**Figure 4:**
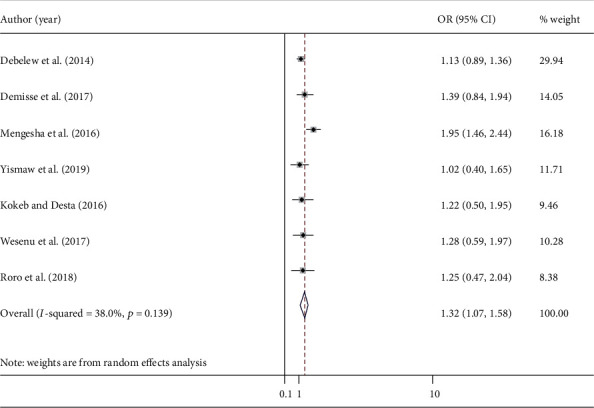
The pooled odds ratio of the association between GA and neonatal mortality.

Seven studies [21, 23, 26, 27, 29, 38, 40] examined the association between gestational age and neonatal mortality. The pooled odds ratio was 1.32 (95% CI: 1.07, 1.58; *I*^2^ = 38%). The current meta-analysis showed that the likelihood of death among preterm neonates was 1.3 times higher as compared with term neonates (Figure 4). Begg's (*p* = 0.37) and Egger's (*p* = 0.7) tests did not reveal significant publication bias.

#### 3.7.2. Residency

**Figure 5:**
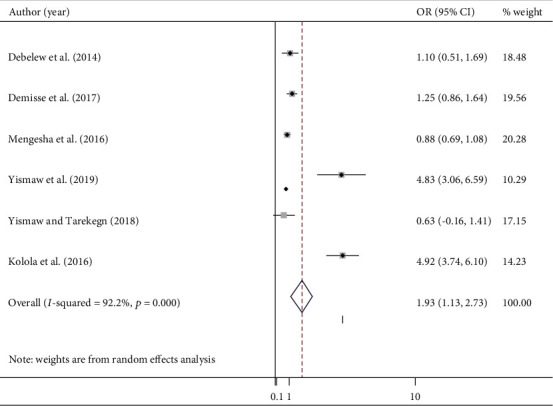
The pooled odds ratio of the association between residency and neonatal mortality.

Six studies [21–23, 26, 38, 41] reported the association between place of residency and neonatal mortality. The pooled odds ratio was 1.93 (95% CI: 1.13, 2.73; *I*^2^ = 92.2%) (Figure 5). The pooled effect of being born in a rural area had a 1.9 times higher risk of mortality than their counterpart. Begg's (*p* = 0.99) and Egger's (*p* = 0.663) tests showed that there was no significant publication bias.

#### 3.7.3. Respiratory Distress Syndromes

**Figure 6:**
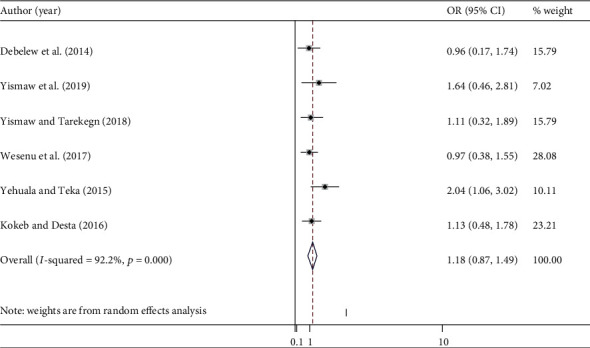
The pooled odds ratio of the association between RDS and neonatal mortality.

According to these findings, those neonates having RDS were also associated with neonatal mortality (Figure 6). Neonates who had RDS were nearly one and half times more likely to die as compared to those who did not have RDS [OR = 1.18 (95% CI: 0.87, 1.49)]. We observed no heterogeneity across the studies (*I*^2^ = 0.00%) with no publication bias (Begg, *p* = 1.000, and Egger, *p* = 0.138).

#### 3.7.4. Neonatal Sepsis

**Figure 7:**
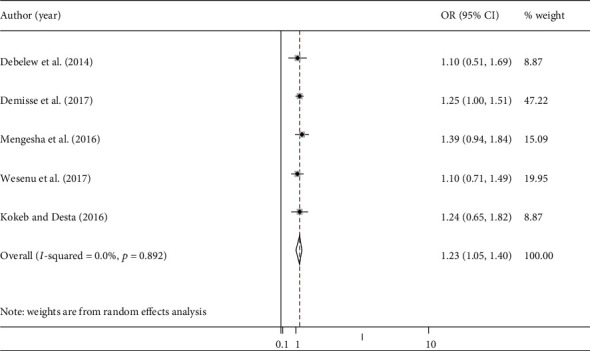
Pooled odds ratio of the association between neonatal sepsis and neonatal mortality.

To assess the association of neonatal sepsis with neonatal mortality, we have included five studies [23, 26, 27, 38, 41]. Patients who had neonatal sepsis had almost one and half times higher chance of getting neonatal death compared to those patients with no sepsis [OR: 1.23 (95% CI: 1.05, 1.40)] (Figure 7). The heterogeneity test showed no evidence of variation across studies (*I*^2^ = 0.0%). Additionally, there were no evidence of publication bias (Begg, *p* = 0.806, and Egger, *p* = 0.511).

#### 3.7.5. Sensitivity Analysis

**Figure 8:**
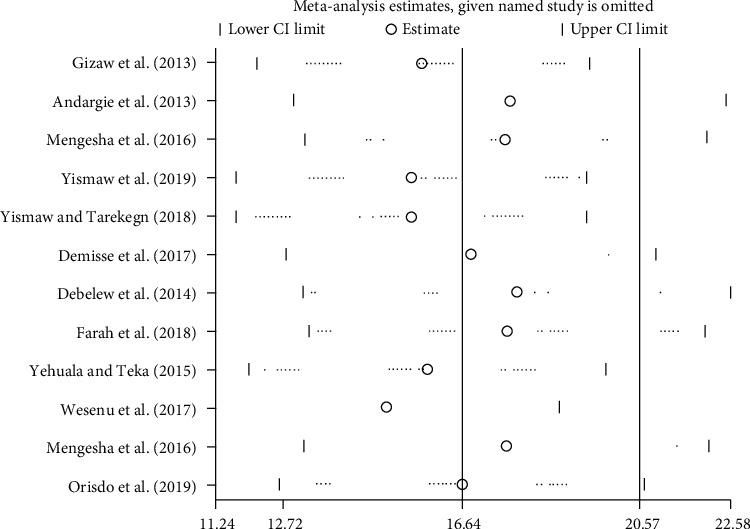
Result of sensitivity analysis of the 24 studies.

Sensitivity analyses using the random effects model revealed that no single study affected the overall prevalence of neonatal mortality (Figure 8).

## 4. Discussion

This systematic review and meta-analysis was aimed at providing comprehensive synthesized evidence on the national burden of neonatal mortality and its major determinant factors in Ethiopia. Our study showed that the national prevalence of neonatal mortality was 16.3%. This is in line with study findings in Cameroon, South Africa, South Sudan, and Mauritanian [42, 43]. However, our finding was lower than the UNICEF national 2016 report [3, 5] and other national reports in Africa [44, 45], Europe, USA, and Central and West Asia [46–48]. This marked difference might be attributed to difference in methodology, sample size, study period, and geographic area. For example, the UNICEF report includes all regions and cities of Ethiopia, but the current study includes studies only five regions. Additionally, some of the previous reports had limited study area instead of nationwide reports. Moreover, the difference in the study period could be a relevant factor as standard or care and treatment modalities change over time. Additionally, though, neonatal health care services in Ethiopia have remained less consolidated; systematic efforts have been gaining momentum relatively recently. Furthermore, the NICU service in our country is not well advanced.

The subgroup analysis indicated that the highest prevalence of mortality was observed in the Amhara region (20.3%), whereas the lowest prevalence was observed in Somalia (5.7%). The possible reason might be that in the Amhara region, five studies were included compared with other regions. Additionally, the sampled populations included in the Amhara region were higher than in other regions.

We also sought gestational age, neonatal sepsis, and residence which significantly predicted neonatal mortality but not respiratory distress syndrome. These studies reported that having neonatal sepsis significantly increased the risk of mortality. Those who had neonatal sepsis were nearly two times at risk of mortality as compared to those who did not have sepsis. This finding is supported by results in developed and developing countries [24, 27, 38, 49]. The possible reason might be due to newborns having many physiologic challenges when adapting to the extrauterine environment which might contribute to common problems like immature immunity, RDS, neurologic, cardiovascular, hematologic, nutritional, gastrointestinal, and poor thermoregulation which further increase the risk of sepsis and mortality. We also found that neonates living in rural areas were more vulnerable to death than their urban counterparts. Neonates from rural households were nearly two times more likely to die as compared to their counterparts. This finding is supported by a study conducted in Washington, Louisiana, and Tennessee [50]. This variance could be due to the fact that rural residents are still relatively disadvantaged in terms of infrastructure, knowledge and awareness difference, and distance from the site. Another possible reason could be that people living in rural areas tend to be poorer than their urban counterparts, a factor known to have an impact on the neonatal outcome.

GA is also another important determinant of neonatal mortality in our meta-analysis. Accordingly, neonates born as preterm were almost one and half times more likely to die than those term neonates. This is supported by a study in Southeast Asian Nations [49], Iran [51], and China [52]. This may be due to the fact that preterm newborns had immaturity of immune systems and body defense mechanisms which help to control newborn infection and disease susceptibility. RDS are also an important risk factor of neonatal mortality although not statistically significant in our meta-analysis. Neonates who had RDS were one times more likely to die as compared to those who did not have RDS. Consistent results have been recorded in our country [24, 53], China [52], and Southeast Asian Nations [49]. This might be due to the fact that babies with RDS do not have a protein called surfactant that keeps small air sacs in the lungs from collapsing which increase the risk of neonatal mortality [54].

Conducting this type of study will act as an input for program planners and policy makers working in the area of neonatal care and also indicate the quality of health care and the welfare of the society. This study is the first meta-analysis in the study area with novel findings. Even though this analysis has delivered valued evidence regarding the level of neonatal mortality and its predictors, there were some limitations, which we address below: this meta-analysis represented only studies reported from five regions of the country. Therefore, the regions may be underrepresented due to the limited number of studies included.

## 5. Conclusion

Despite the variation across regions, neonatal mortality is a major public problem in Ethiopia. Gestational age, neonatal sepsis, RDS, and residence were an important predictor of neonatal mortality. Therefore, particular emphasis shall be given to the rural communities. Additionally, the government should have to strengthen any service related with reducing the neonatal mortality like expanding NICU all over the country. The health care professionals should also give a particular emphasis and work on early diagnosis and treatment of preterm neonates with sepsis and RDS. In addition, this study may help policy makers and program managers to design interventions on reducing the rate of neonatal mortality.

## Figures and Tables

**Table 1:** Characteristics of the included studies.

Authors/year	Area	Region	Study design	Total sample	Response rate	Prevalence
Gizaw et al./2013 [28]	Butajira	SNNP	Cohort	1055	83.9	26%
Andargie et al./2013 [25]	Gondar	Amhara	Cross-sectional	1752	100	5%
Mengesha et al./2016 [37]	Tigray	Tigray	Cohort	1162	99.14	6%
Yismaw et al./2019 [21]	Gondar	Amhara	Cohort	516	100	29%
Yismaw and Tarekegn/2018 [22]	Gondar	Amhara	Cross-sectional	516	100	28.8%
Demisse et al./2017 [23]	Gondar	Amhara	Cross-sectional	769	100	14.3%
Debelew et al./2014 [26]	Jimma	Oromia	Cohort	3604	96.1	3.2%
Farah et al./2018 [39]	Somali	Somali	Cohort	792	100	5.7%
Yehuala and Teka/2015 [24]	Gondar	Amhara	Cohort	485	100	25%
Wesenu et al./2017 [27]	Jimma	Oromia	Cohort	490	100	34.5%
Mengesha et al./2016 [38]	Mekelle	Tigray	Cohort	1162	99.04	5.8%
Orsido et al./2019 [30]	Weliata	SNNP	Cohort	964	100	16.5%

**Table 2:** The prevalence of neonatal mortality by region, study design, and sample size.

Variables	Characteristics	Included studies	Sample size	Prevalence (95% CI)
Regions	Oromia	2	3953	18.8 (11.9, 49.4)
	Amhara	5	4038	20.3 (9.6, 31.1)
	Tigray	2	2304	5.9 (4.9, 6.8)
	SNNP	2	1850	18.2 (11.9, 30.5)
	Somali	1	792	9.1 (23.1, 8.9)
Study design	Cohort	8	8936	16.6 (11.2, 21.9)
	Cross-sectional	3	3037	15.9 (3.9, 27.8)
By included sample size	≥800	5	8405	8.9 (4.9, 12.9)
	<800	6	3568	22.7 (12.8, 32.7)
Overall		11	12397	16.3 (11.9, 20.7)

**Table 3:** Metaregression analysis of neonatal sepsis with heterogeneity of neonatal mortality.

Heterogeneity source	Coefficients	Std. err.	*p* value
Publication year	-1.243	4.738	0.64
Sample size	0.01800	0.290	0.86

## Data Availability

The data used to support the findings of this study are available within the article and its supplementary information files.

## Abbreviations

CI: Confidence interval
GA;: Gestational age
OR: Odds ratio
NMR: Neonatal mortality rate
PRISMA: Preferred Reporting Items for Systematic Reviews and Meta-Analyses
RDS: Respiratory distress syndromes
SNNP: Southern Nations, Nationalities, and Peoples
WHO: World Health Organization.

## Supplementary Materials

Supplementary Materials Supplementary file 1: methodological quality assessment of cross-sectional studies using the modified Newcastle-Ottawa Scale (NOS).
