# Supplementary material for: The Magnitude of Neonatal Mortality and Its Predictors in Ethiopia: A Systematic Review and Meta-Analysis
Source: Int J Pediatr. 2021 Feb 17;2021:7478108. doi: 10.1155/2021/7478108 (PMC7906817; doi:10.1155/2021/7478108)
Supplement: Supplementary Materials — Supplementary file 1: methodological quality assessment of cross-sectional studies using the modified Newcastle-Ottawa Scale (NOS). [file 7478108.f1.docx]

**Supplementary files for methodological quality assessment**

| **First author** | Criteria |  |  |  |  |  |  |  |  |
| --- | --- | --- | --- | --- | --- | --- | --- | --- | --- |
|  | **Selection** |  |  |  | **Comparability** |  | **Outcome** |  |  |
|  | **Representativ** | **Sample size** | **Non –** | **Ascertainment of** | **The study** | **The study** | **Assessment of** | **Statistical test** | **Total** |
|  | **eness of the** |  | **responder** | **exposure/risk** | **controls for the** | **control for** | **the outcome** |  | **score** |
|  | **sample** |  | **s** | **factor** | **most important** | **any** |  |  | **(10)** |
|  |  |  |  |  | **factor** | **additional** |  |  |  |
|  |  |  |  |  |  | **factor** |  |  |  |
| Gizaw et al.2013 | A* | **B*** | **B*** | **A*** | **-** | **B*** | A* | **A*** | **7** |
| El Saghier EO et al | B* | **B*** | **A*** | **A*** | **A*** | **-** | A* | **A*** | **7** |
| [22] |  |  |  |  |  |  |  |  |  |
| **Andargie et** | **A*** |  |  |  |  |  |  |  |  |
| **al.,2013** |  | **A*** | **A*** | **A*** | **A*** | **-** | A* | **A*** | **7** |
|  | A* | **B*** | **B*** | **A*** | **-** | **B*** | A* | **A*** | **7** |
| **Mengesha et** |  |  |  |  |  |  |  |  |  |
| **al.,2016** |  |  |  |  |  |  |  |  |  |
| **Yismaw et al.,2019** | B* | **B*** | **A*** | **A*** | **A*** | **-** | A* | **A*** | **7** |
|  |  |  |  |  |  |  |  |  |  |
| **Yismaw and** |  |  |  |  |  |  |  |  |  |
| **Tarekegn etal.,2018** | **B*** | **A*** | **A*** | **B*** | **A*** | **A*** | A* | **A*** | **7** |
|  |  |  |  |  |  |  |  |  |  |
| **Demisse et al**.,2017 | **A*** | **A*** | **A*** | **A*** | **A*** | **B*** | A* | **A*** | **7** |
|  |  |  |  |  |  |  |  |  |  |
| **Farah et al.,2018** | B* | **B*** | **A*** | **A*** | **A*** | **-** | A* | **A*** | **7** |
|  |  |  |  |  |  |  |  |  |  |
| **Yehuala and Teka** |  |  |  |  |  |  |  |  |  |
| **etal.,2015** | A* | **A*** | **-** | **A*** | **A*** | **-** | A* | **A*** | **6** |
|  |  |  |  |  |  |  |  |  |  |
| **Wesenu et al.,2017** | **A*** | **A*** | **A*** | **B*** | **A*** | **A*** | A* | **A*** | **8** |
| **Mengesha et** |  |  |  |  |  |  |  |  |  |
| **al.,2016** | A* | **B*** | **B*** | **A*** | **-** | **B*** | A* | **A*** | **7** |
|  |  |  |  |  |  |  |  |  |  |

| **Orsido et al.,2019** | **A*** | **A*** | **A*** |  | **B*** | **B*** | A* | **A*** | **6** |
| --- | --- | --- | --- | --- | --- | --- | --- | --- | --- |
|  |  |  |  |  |  |  |  |  |  |

*Note: from each item account point. (Accept the study if total score ≥5)*

**Selection:** (Maximum 5 stars)

1. Representativeness of the sample: a) Truly representative of the average in the target population. * (all subjects or random sampling) .b) Somewhat representative of the average in the target population. * (nonrandom sampling) .c) Selected group of users.d) No description of the sampling strategy.
2. Sample size:a) Justified and satisfactory. *.b) Not justified.
3. Non-respondents: a) Comparability between respondents and non-respondents characteristics is

established, and the response rate is satisfactory. * .b) The response rate is unsatisfactory, or the comparability between respondents and non-respondents is unsatisfactory. c) No description of the response rate or the characteristics of the responders and

the non-responders.

1. Ascertainment of the exposure (risk factor): a) validated measurement tool. ** .b) Non-validated measurement tool, but the tool is available or described.* c) No description of the measurement tool.

**Comparability:** (Maximum 2 stars)

1. The subjects in different outcome groups are comparable, based on the study design or analysis. Confounding factors are controlled. a) The study controls for the most important factor (select one). * b) The study control for any additional factor. *

**Outcome:** (Maximum 3 stars)

1. Assessment of the outcome: a) Independent blind assessment. **,b) Record linkage. **,c) Self report. *,d) No description.
2. Statistical test:a) The statistical test used to analyze the data is clearly described and appropriate, and the measurement of the association is presented, including confidence intervals and the probability level (p value). *,b) The statistical test is not appropriate, not described or incomplete

**Supplementary file 1:**: Methodological quality assessment of cross-sectional and cohort studies using modified Newcastle - OttawaScale (NOS)

| **First author, publication year** | **Criteria** |  |  |  |  |  |  |  |
| --- | --- | --- | --- | --- | --- | --- | --- | --- |
|  | **Selection** |  |  | **Comparability** |  | **Outcome** |  |  |
|  | **Representativeness of the** | **Non –** | **Ascertainme** | **The study** | **The study** | **Assessment** | **Statist** | **Total** |
|  | **sample** | **respon** | **nt of** | **controls for** | **control for** | **of the** | **ical** | **score** |
|  |  | **dents** | **exposure** | **the most** | **any** | **outcome** | **test** |  |
|  |  |  |  | **important** | **additional** |  |  |  |
|  |  |  |  | **factor** | **factor** |  |  |  |
| Gizaw et al.2013 | **1** | **1** | **1** | **1** | **1** | **1** | **1** | **7** |
|  |  |  |  |  |  |  |  |  |
| **Andargie et al.,2013** | **1** | **1** | **1** | **1** | **1** | **1** | **1** | **7** |
|  |  |  |  |  |  |  |  |  |
| **Mengesha et al.,2016** | **1** | **1** | **1** | **1** | **1** | **1** | **1** | **7** |
| **Yismaw et al.,2019** | **1** | **0** | **1** | **1** | **1** | **1** | **1** | **6** |

| **Yismaw and Tarekegn** | **1** | **1** | **1** | **1** | **1** | **1** | **1** | **7** |
| --- | --- | --- | --- | --- | --- | --- | --- | --- |
| **etal.,2018** |  |  |  |  |  |  |  |  |
|  |  |  |  |  |  |  |  |  |
| **Demisse et al**.,2017 | **1** | **1** | **1** | **1** | **1** | **1** | **1** | **7** |
| **Debelew et al.** 2014 | 1 | **1** | **1** | **1** | **1** | 1 | **1** | **5** |
|  |  |  |  |  |  |  |  |  |
| **Farah et al.,2018** | 1 | **1** | **1** | **1** | **1** | 1 | **1** | **7** |
| **Yehuala and Teka etal.,2015** | 1 | **1** | **1** | **1** | **1** | 1 | **1** | **7** |
|  |  |  |  |  |  |  |  |  |
| **Wesenu et al.,2017** | 1 | **0** | **1** | **0** | **1** | 1 | **1** | **6** |
|  |  |  |  |  |  |  |  |  |
| **Mengesha et al.,2016** | 1 | **1** | **1** | **1** | **1** | 1 | **1** | **7** |
|  |  |  |  |  |  |  |  |  |
| Orsido et al.,2019 | 1 | **0** | **1** | **0** | **1** | 1 | **1** | **5** |
|  |  |  |  |  |  |  |  |  |

*Note: each item account 1 point. (Accept the study if total score ≥5)*

Non-respondents:

a) Comparability between respondents and non-respondents characteristics is established, and the response rate is satisfactory. **^ ^**

Representativeness of the sample:

1. Truly representative of the average in the target population. **^^** (all subjects or random sampling)
2. Somewhat representative of the average in the target population. **^^** (non-random sampling)

Comparability: The subjects in different outcome groups are comparable, based on the study design or analysis. Confounding factors are controlled. a) The study controls for the most important factor (select one).**^^**

b) The study control for any additional factor. **^^**

Ascertainment of exposure based on validated measurement tool**^ ^**or non-validated measurement tool, but the tool is available or described OR No description of the measurement tool.

Outcome:Independent blind assessment**^^**, record linkage**^^**, self-report or no description.

The statistical test used to analyse the data is clearly described and appropriate, and the measurement of the association is presented, including confidence intervals and the probability level (p value)**^^** or the statistical test is not appropriate, not described or incomplete.
